# Deciphering the roles of macrophages in developmental and inflammation stimulated lymphangiogenesis

**DOI:** 10.1186/2045-824X-4-15

**Published:** 2012-09-03

**Authors:** Natasha L Harvey, Emma J Gordon

**Affiliations:** 1 Division of Haematology, Centre for Cancer Biology, SA Pathology, Adelaide, Australia; 2 Department of Cardiology, Yale University School of Medicine, New Haven, CT, USA

**Keywords:** Lymphangiogenesis, Macrophages, Monocytes, Development, Inflammation, VEGF-C, VEGF-D

## Abstract

Lymphatic vessels share an intimate relationship with hematopoietic cells that commences during embryogenesis and continues throughout life. Lymphatic vessels provide a key conduit for immune cell trafficking during immune surveillance and immune responses and in turn, signals produced by immune lineage cells in settings of inflammation regulate lymphatic vessel growth and activity. In the majority of cases, the recruitment and activation of immune cells during inflammation promotes the growth and development of lymphatic vessels (lymphangiogenesis) and enhances lymph flow, effects that amplify cell trafficking to local lymph nodes and facilitate the mounting of effective immune responses. Macrophages comprise a major, heterogeneous lineage of immune cells that, in addition to key roles in innate and adaptive immunity, perform diverse tasks important for tissue development, homeostasis and repair. Here, we highlight the emerging roles of macrophages in lymphangiogenesis, both during development and in settings of pathology. While much attention has focused on the production of pro-lymphangiogenic stimuli including VEGF-C and VEGF-D by macrophages in models of inflammation including cancer, there is ample evidence to suggest that macrophages provide additional signals important for the regulation of lymphatic vascular growth, morphogenesis and function.

## The many faces of macrophages

Macrophages encompass a phenotypically heterogeneous population of cells that play a rapidly expanding catalogue of roles during development, homeostasis and disease [1-3]. While perhaps best recognised for the key roles they fulfil in innate and adaptive immunity, macrophages (literally “large eaters”, due to their phagocytic capabilities) also provide apoptosis inducing stimuli important for tissue remodeling and maturation [4-6], cues that instruct organ patterning and morphogenesis [7-12] and signals important for tissue regeneration and repair [13-15].

Macrophage diversity is obvious both in the embryo and the adult. In the embryo, macrophage subtypes can be distinguished on the basis of differential expression of markers including lymphatic vessel endothelial hyaluronan receptor (LYVE-1) and the angiopoietin receptor Tie2 [16,17]. In the adult, distinct populations of circulating monocytes are categorised as “inflammatory” or “resident” monocytes based on the expression of markers including Gr1/Ly6C and the chemokine receptors CX_3_CR1 and CCR2 [18]. Tie2 has been reported to mark a distinct lineage of monocytes, termed Tie2-expressing monocytes (TEMs), that are recruited to the tumor microenvironment where they promote the growth of new blood vessels [19]. In response to stimuli encountered, monocytes may be induced to differentiate into macrophage subtypes including classically activated, “M1” inflammatory macrophages or alternatively activated, “M2” regulatory and wound healing macrophages. There is little doubt, however, that the M1 and M2 classification oversimplifies the extent of macrophage heterogeneity [20,21]. Mature, specialist macrophages found in adult tissues include Kupffer cells of the liver that clear spent erythrocytes from the circulation, microglia in the central nervous system that regulate neural development and osteoclasts in the bone marrow important for bone remodeling [1,3].

Recent work has revealed that macrophages play key roles during vascular development; these include directing regression of the hyaloid blood vascular plexus [4,5,22], facilitating the anastomosis of sprouting blood vessels [10] and patterning the retinal vasculature [23,24]. Furthermore, macrophages have been shown to promote neo-vascularization in settings of inflammation and wound repair by producing pro-angiogenic growth factors, chemokines and proteases [19,25-27]. In this review, we focus on the roles that macrophages play in lymphatic vascular growth and development (lymphangiogenesis), both during development and in disease.

## Developmental origins of macrophages and lymphatic vessels

The developmental origins of macrophage subsets are being progressively unravelled, with genetic lineage tracing studies providing answers to longstanding questions regarding progenitor cell origin and the differentiation potential of various monocyte/macrophage populations. Current data suggest that maternally derived macrophages are the first to appear in the mouse embryo at approximately embryonic day (E) 7.5 [28]. Subsequently, macrophages originating in the yolk sac (E8) and from definitive hematopoietic progenitor cells arising in the embryonic aorta-gonad-mesonephros (AGM) (E10.0) and foetal liver develop (E10.5-onwards) [28]. After birth, the major site of hematopoiesis is the bone marrow. Recent evidence supports the concept that mature macrophages in the adult are derived from distinct progenitor pools; microglia in the brain appear to be descendents of primitive myeloid progenitors that arise prior to E8 [29], while circulating monocytes that give rise to the majority of tissue macrophages are derived from definitive hematopoietic stem cells.

Construction of the lymphatic vasculature is initiated once the major arteries (dorsal aortae) and veins (cardinal veins) have been established in the embryo, originating from the cardinal veins following the onset of expression of the Prox1 transcription factor in a polarised population of venous endothelial cells at ~ E9.5 [30]. Prox1-positive lymphatic endothelial progenitor cells exit the cardinal veins via sprouting and ballooning mechanisms to form lymph sacs and the superficial lymphatic vascular plexus [30,31], a process that is dependent on vascular endothelial growth factor C (VEGF-C) [32]. The initiation of Prox1 expression in lymphatic endothelial progenitor cells in the cardinal veins signifies lymphatic endothelial cell fate commitment and is dependent on the activity of Sox18 and CoupTFII transcription factors [33,34]. Once established, the lymphatic vasculature regulates tissue fluid homeostasis, immune cell trafficking and the absorption of dietary fats [35,36].

**Figure 1:**
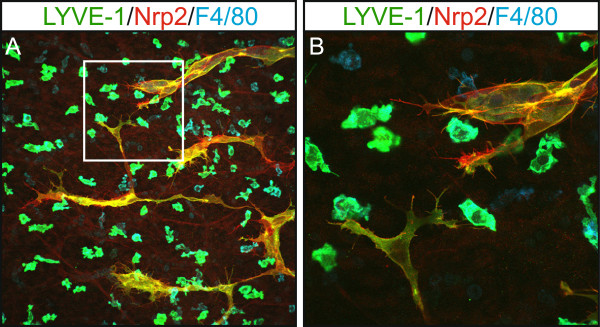
**LYVE-1 positive macrophages share an intimate association with growing lymphatic vessels in embryonic mouse skin.** Whole mount immunostaining of E14.5 skin illustrating the close association of lymphatic vessel filopodia (Nrp2-positive, LYVE-1-positive) with dermal macrophages (F4/80-positive, LYVE-1-positive). Area boxed in panel **A** is shown at higher magnification in panel **B**.

Work in a variety of vertebrate models has suggested that mesodermal cells, including those of the macrophage lineage, might contribute to genesis of the lymphatic vasculature during development by comprising a pool of lymphatic endothelial progenitor cells [37-40]. These studies have suggested that mesenchymal “lymphangioblasts” expressing both LEC (Prox1/LYVE-1) and macrophage markers (LYVE-1, CD45, F4/80) integrate to growing lymphatic vessels in the developing embryo [38,40]. In contrast, work from others has concluded that the vast majority of embryonic lymphatic endothelial cells are derived from the venous progenitor pool, with no evidence to support the concept of monocytes or macrophages giving rise to lymphatic endothelial cells [17,41]. These discrepancies may potentially be a result of differences between the vertebrate models employed in these studies, or of the techniques utilized to assess macrophage incorporation into lymphatic vessels (marker expression versus lineage tracing). In fact, embryonic LYVE-1-positive macrophages have been found to share an intimate spatial association with embryonic lymphatic vessels (Figure 1) and in some cases appear incorporated into the wall of developing lymphatic vessels, but lineage tracing studies in the mouse embryo have not detected Prox1 expression in LYVE-1-positive, myeloid derived cells [17]. This data suggests that these cells retain a macrophage identity even when resident in lymphatic vessels. Gene expression profiling of embryonic dermal LYVE-1-positive macrophages revealed a close resemblance to TEMs [16,17], a population of macrophages that not only play key roles in tumor stimulated angiogenesis, but are important for blood vascular development [10,16,19,42]. These data suggest that LYVE-1-positive macrophages may be important for morphogenesis or remodelling of the lymphatic vasculature. Studies utilising real time imaging will be required to discern whether LYVE-1-positive macrophages assume localisation in the walls of lymphatic vessels in order to perform an immune surveillance role, whether they transit through the lymphatic endothelium while patrolling the embryo, or whether they fulfil roles important for lymphatic vascular development.

## Macrophages as a source of growth and patterning signals in developmental lymphangiogenesis

While it seems unlikely that monocytes/macrophages comprise a lymphatic endothelial progenitor cell pool during embryogenesis, recent work from a number of groups has identified an important role for macrophages in patterning the lymphatic vasculature. Akin to the role identified for macrophages in mediating the anastomosis of sprouting blood vessels in the embryonic brain [10] and postnatal retina [23], work from Kubota and colleagues suggested that LYVE-1-positive macrophages regulate density of the lymphatic vasculature in selected postnatal tissues [43]. Analysis of *Csf1*^*op/op*^ mice that lack a key growth factor for macrophage development, Csf1, and are therefore severely depleted of macrophages [44], revealed diminished lymphatic vessel density in the postnatal trachea [43]. Moreover, depletion of macrophages using antibodies to c-fms (the receptor for Csf1, expressed by macrophages), or the small molecule c-fms tyrosine kinase inhibitor Ki20227, between postnatal day (P)8 and P15, resulted in reduced lymphatic vessel branching in the trachea and ears of treated mice. However, these studies found no evidence of perturbed embryonic lymphangiogenesis in *Csf1*^*op/op*^ mice and lymphatic vascular patterning appeared normal in surviving *Csf1*^*op/op*^ mice at 3 months of age [43]. These observations suggest a temporal window of macrophage dependence, or potential tissue specific roles, for macrophages in developmental lymphangiogenesis.

Work from Bohmer and colleagues demonstrated that embryonic dermal macrophages expressing the tyrosine kinase Syk closely resemble TEMs and express high levels of pro-lymphangiogenic molecules including vascular endothelial growth factor C (VEGF-C), vascular endothelial growth factor D (VEGF-D), fibroblast growth factor 2 (FGF2), matrix metalloprotease 2 (MMP-2) and matrix metalloprotease 9 (MMP-9) [45]. In the absence of Syk, an increased number of these pro-lymphangiogenic monocytes/macrophages expressing elevated levels of growth factors and chemokines accumulated in skin and as a result, *Syk*^*−/−*^ embryos displayed hyperplastic dermal lymphatic vessels. These data suggest that dermal Syk-expressing macrophages have the potential to promote embryonic lymphangiogenesis via the production of a number of pro-lymphangiogenic stimuli. In agreement with these studies, Gordon and colleagues found that primary embryonic dermal macrophages promoted the proliferation of primary embryonic dermal LEC when cultured together *ex vivo*[17]. Intriguingly though, the dermal lymphatic vasculature of embryonic *PU.1*^*−/−*^[46] and *Csf1r*^*−/−*^[47] macrophage deficient mice was found to be hyperplastic, rather than hypoplastic [17]. These data suggest that while dermal macrophages have the capacity to promote lymphangiogenesis via the production of pro-lymphangiogenic growth factors including VEGF-C and VEGF-D, they may also act to restrain lymphatic endothelial cell proliferation during development. In contrast to the dermal lymphatic vasculature, Gordon and colleagues found that the jugular lymph sacs of embryonic *PU.1*^*−/−*^ mice were smaller than their wild-type littermates, providing further evidence that macrophages may play distinct, tissue specific roles during developmental lymphangiogenesis [17]. Definitively dissecting the relative contribution of macrophage-derived signals during developmental lymphangiogenesis will rely on the generation of conditional knockout mice in which genes of interest are inactivated in a macrophage selective manner.

## Macrophages in inflammation and tumor-stimulated lymphangiogenesis

The roles played by macrophages in pathological lymphangiogenesis are rapidly gaining recognition. The induction of lymphangiogenesis during wound healing and in many pathological settings normally acts to resolve inflammation and edema; in models of infection and acute inflammation, increased lymphangiogenesis has been associated with elevated lymph flow, enhanced immune cell trafficking to draining lymph nodes and the resolution of tissue inflammation and edema [48-50]. While this is advantageous in the majority of settings, lymphangiogenesis induced following organ transplantation has been shown to aid alloimmunization and thereby promote the rejection of kidney, corneal and pancreatic islet transplants [51-53]. Understanding how lymphangiogenesis is regulated during inflammation thereby stands to advance the development of new therapeutics able to promote or ablate lymphangiogenesis dependent on the pathological setting.

**Figure 2:**
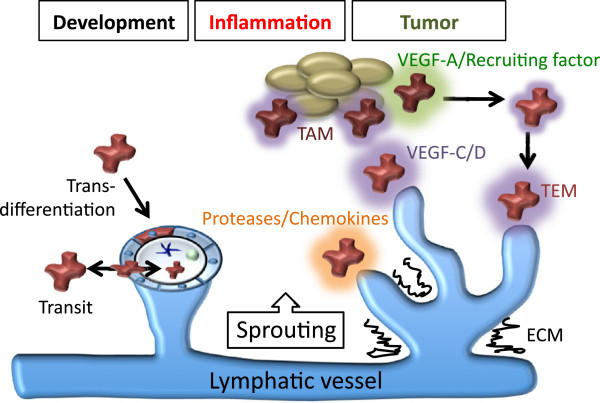
**A model depicting the roles of macrophages in lymphangiogenesis.** Macrophages have been proposed to contribute to lymphangiogenesis by acting as a source of lymphatic endothelial progenitor cells which incorporate into growing vessels, or by providing growth and patterning signals such as VEGF-A and VEGF-C/D, that stimulate the growth and/or function of the lymphatic vasculature. Lineage tracing studies have suggested that macrophages do not act as lymphatic endothelial progenitor cells, yet they share a close spatial localization with lymphatic vessels during development and express the lymphatic marker LYVE-1. These macrophages may assume localization in the walls of lymphatic vessels in order to perform an immune surveillance role, or be transiting through the lymphatic endothelium. Tumor associated macrophages (TAMs) and Tie2-expressing monocytes (TEMs) promote lymphangiogenesis by liberating proteases important for growth factor activation/matrix remodeling, producing chemokines that degrade the surrounding extracellular matrix (ECM), and recruiting additional inflammatory cells.

Macrophages have been demonstrated to drive lymphangiogenesis in models of inflammation including bacterial infection [48,49,54], wound healing [55,56], organ transplant [57,58], rheumatoid arthritis [59], pancreatic islet inflammation/diabetes [60] and atopic dermatitis [61]. Macrophages also appear to be key players in salt-induced hypertension, where macrophage derived VEGF-C is important for inducing lymphangiogenesis as a buffering mechanism to deal with increased interstitial fluid accumulation [62]. At least two mechanisms by which cells of the monocyte/macrophage lineage promote neo-lymphangiogenesis have been proposed; trans-differentiation to lymphatic endothelial cells [55,57,63-65] and production of pro-lymphangiogenic stimuli including VEGF-C, VEGF-D and VEGF-A [48,49,54,66-68] (Figure 2). The relative contribution that macrophages provide to inflammation-stimulated lymphangiogenesis has been illustrated in studies of macrophage deficient mice [43] and by the depletion of macrophages using clodronate liposomes [49,67], c-fms inhibition [43] or VEGF-A inhibition [49,67,69]. VEGF-C and/or VEGF-D appear to be critical for the pro-lymphangiogenic activity of macrophages in the majority of models studied; blockade of VEGF-C and VEGF-D activity using soluble VEGFR-3 or VEGFR-3 neutralising antibodies has been demonstrated to inhibit macrophage driven lymphangiogenesis [48,49,52,68]. Recent work has suggested that the extracellular matrix protein thrombospondin-1 (TSP-1) acts as an endogenous inhibitor of corneal lymphangiogenesis via acting on macrophages; ligation of CD36 on the macrophage cell surface by Tsp-1 was shown to negatively regulate VEGF-C and VEGF-D production. In the absence of Tsp-1, and in mice deficient in CD36, precocious lymphangiogenesis is induced in the cornea [70]. In addition to VEGF family members, macrophages are a source of proteases including MMP-2 and MMP-9 [1,26,45] that promote growth factor activation and matrix remodelling, as well as cytokines and chemokines that recruit additional cells of the immune system. Immune cells including granulocytes, B and T lymphocytes have also been demonstrated to have pro- and anti lymphangiogenic activities [48,71-73] and their capacity to regulate lymphangiogenesis should be taken into account when looking at the “big picture” of inflammation.

Many studies have shown that the growth of lymphatic vessels in the tumor microenvironment facilitates tumor metastasis [74]. Tumor-stimulated lymphangiogenesis often results in the formation of abnormal, leaky lymphatic vessels, a feature that provides metastatic tumor cells with ready access to the lymphatic vasculature [75,76]. Macrophage recruitment to tumors has been demonstrated to promote lymphangiogenesis in a variety of mouse tumor models [43,66,68,77,78] and tumor associated macrophages (TAMs) have been linked with increased peri-tumoral lymphangiogenesis and metastasis in human cancers including breast cancer [79], cervical cancer [66], squamous cell carcinoma [80] and advanced colorectal cancer [81]. In a mouse model of osteosarcoma, inhibition of Csf1 diminished macrophage recruitment to the tumor environment, suppressed tumor angiogenesis and lymphangiogenesis and reduced tumor metastasis [43]. Similarly, in a model of urinary bladder cancer, depletion of TAMs with clodronate liposomes and suppression of lymphangiogenesis with soluble VEGFR-3 inhibited lymphangiogenesis and tumor metastasis [77]. Studies such as these suggest that therapeutics designed to block macrophage influx or inhibit macrophage activity might provide valuable anti-tumor and anti-metastatic agents. In addition to promoting the growth of new lymphatic vessels in the vicinity of tumors, macrophage derived pro-lymphangiogenic growth factors including VEGF-C and VEGF-D may act downstream of the initial lymphatics on collecting vessels to promote their dilation and capacity for lymph flow, thereby facilitating metastatic tumor cell transport [35,82]. TAMs may also contribute to lymphangiogenesis associated pathologies in addition to aiding metastasis. When a metastatic human ovarian cancer cell line was transplanted into mice, Jeon and colleagues found that tumor progression was associated with the development of chylous ascites due to a profound dysfunctional lymphangiogenic response [68]. Blockade of VEGF-C/-D with soluble VEGFR-3 and of VEGF-A signaling with VEGF-Trap prevented the formation of chylous ascites, implicating macrophage derived VEGF-C/-D in ascites development due to the failure of aberrant lymphatic vessels to mediate fluid clearance from the peritoneal cavity [68]. This study has implications for the treatment of ascites in ovarian cancer patients.

## Perspectives and future directions

A growing body of data now cements “regulation of lymphangiogenesis during development and disease” together with the plethora of important roles that macrophages play in tissue morphogenesis, homeostasis, repair and immunity. Many questions remain to be answered before we will completely understand how macrophages regulate lymphangiogenesis. Current topical questions include: What is the relative contribution of macrophage derived VEGF-C and -D to embryonic and inflammation stimulated lymphangiogenesis? What signals in addition to VEGF family members do macrophages provide that control lymphatic vascular growth and morphogenesis? Do different macrophage subtypes differ with respect to their pro- or anti- lymphangiogenic activity? The generation of new experimental tools including genetically modified mice and agents able to specifically track and manipulate macrophage sub-types will be important for advancing these studies. Though the blockade of macrophages and/or macrophage derived factors poses an attractive strategy for anti-inflammatory and anti-tumor therapies, it will first be important to determine the precise roles of these intriguing cells in developmental and pathological lymphangiogenesis.

## Competing interests

The authors declare that they have no competing interests.

## Author contributions

NH and EG wrote the manuscript and prepared the figures. All authors read and approved the final manuscript.

## References

[B1] Pollard JW Trophic macrophages in development and disease Nat Rev Immunol 2009 9 259 270 10.1038/nri2528 19282852 PMC3648866

[B2] Nucera S Biziato D De Palma M The interplay between macrophages and angiogenesis in development, tissue injury and regeneration Int J Dev Biol 2011 55 495 503 10.1387/ijdb.103227sn 21732273

[B3] Stefater JA Ren S Lang RA Duffield JS Metchnikoff's policemen: macrophages in development, homeostasis and regeneration Trends Mol Med 2011 17 743 752 10.1016/j.molmed.2011.07.009 21890411 PMC3225647

[B4] Lang RA Bishop JM Macrophages are required for cell death and tissue remodeling in the developing mouse eye Cell 1993 74 453 462 10.1016/0092-8674(93)80047-I 8348612

[B5] Lobov IB Rao S Carroll TJ Vallance JE Ito M Ondr JK Kurup S Glass DA Patel MS Shu W WNT7b mediates macrophage-induced programmed cell death in patterning of the vasculature Nature 2005 437 417 421 10.1038/nature03928 16163358 PMC4259146

[B6] Marin-Teva JL Dusart I Colin C Gervais A van Rooijen N Mallat M Microglia promote the death of developing Purkinje cells Neuron 2004 41 535 547 10.1016/S0896-6273(04)00069-8 14980203

[B7] Van Nguyen A Pollard JW Colony stimulating factor-1 is required to recruit macrophages into the mammary gland to facilitate mammary ductal outgrowth Dev Biol 2002 247 11 25 10.1006/dbio.2002.0669 12074549

[B8] Rae F Woods K Sasmono T Campanale N Taylor D Ovchinnikov DA Grimmond SM Hume DA Ricardo SD Little MH Characterisation and trophic functions of murine embryonic macrophages based upon the use of a Csf1r-EGFP transgene reporter Dev Biol 2007 308 232 246 10.1016/j.ydbio.2007.05.027 17597598

[B9] Chua AC Hodson LJ Moldenhauer LM Robertson SA Ingman WV Dual roles for macrophages in ovarian cycle-associated development and remodelling of the mammary gland epithelium Development 2010 137 4229 4238 10.1242/dev.059261 21068060

[B10] Fantin A Vieira JM Gestri G Denti L Schwarz Q Prykhozhij S Peri F Wilson SW Ruhrberg C Tissue macrophages act as cellular chaperones for vascular anastomosis downstream of VEGF-mediated endothelial tip cell induction Blood 2010 116 829 840 10.1182/blood-2009-12-257832 20404134 PMC2938310

[B11] Banaei-Bouchareb L Gouon-Evans V Samara-Boustani D Castellotti MC Czernichow P Pollard JW Polak M Insulin cell mass is altered in Csf1op/Csf1op macrophage-deficient mice J Leukoc Biol 2004 76 359 367 10.1189/jlb.1103591 15178709

[B12] Van Wesenbeeck L Odgren PR MacKay CA D'Angelo M Safadi FF Popoff SN Van Hul W Marks SC The osteopetrotic mutation toothless (tl) is a loss-of-function frameshift mutation in the rat Csf1 gene: Evidence of a crucial role for CSF-1 in osteoclastogenesis and endochondral ossification Proc Natl Acad Sci USA 2002 99 14303 14308 10.1073/pnas.202332999 12379742 PMC137879

[B13] Arnold L Henry A Poron F Baba-Amer Y van Rooijen N Plonquet A Gherardi RK Chazaud B Inflammatory monocytes recruited after skeletal muscle injury switch into antiinflammatory macrophages to support myogenesis J Exp Med 2007 204 1057 1069 10.1084/jem.20070075 17485518 PMC2118577

[B14] Lin SL Li B Rao S Yeo EJ Hudson TE Nowlin BT Pei H Chen L Zheng JJ Carroll TJ Macrophage Wnt7b is critical for kidney repair and regeneration Proc Natl Acad Sci USA 2010 107 4194 4199 10.1073/pnas.0912228107 20160075 PMC2840080

[B15] Duffield JS Forbes SJ Constandinou CM Clay S Partolina M Vuthoori S Wu S Lang R Iredale JP Selective depletion of macrophages reveals distinct, opposing roles during liver injury and repair J Clin Invest 2005 115 56 65 15630444 10.1172/JCI22675 PMC539199

[B16] Pucci F Venneri MA Biziato D Nonis A Moi D Sica A Di Serio C Naldini L De Palma M A distinguishing gene signature shared by tumor-infiltrating Tie2-expressing monocytes, blood "resident" monocytes, and embryonic macrophages suggests common functions and developmental relationships Blood 2009 114 901 914 10.1182/blood-2009-01-200931 19383967

[B17] Gordon EJ Rao S Pollard JW Nutt SL Lang RA Harvey NL Macrophages define dermal lymphatic vessel calibre during development by regulating lymphatic endothelial cell proliferation Development 2010 137 3899 3910 10.1242/dev.050021 20978081 PMC3049282

[B18] Geissmann F Jung S Littman DR Blood monocytes consist of two principal subsets with distinct migratory properties Immunity 2003 19 71 82 10.1016/S1074-7613(03)00174-2 12871640

[B19] De Palma M Venneri MA Galli R Sergi Sergi L Politi LS Sampaolesi M Naldini L Tie2 identifies a hematopoietic lineage of proangiogenic monocytes required for tumor vessel formation and a mesenchymal population of pericyte progenitors Cancer Cell 2005 8 211 226 10.1016/j.ccr.2005.08.002 16169466

[B20] Mosser DM Edwards JP Exploring the full spectrum of macrophage activation Nat Rev Immunol 2008 8 958 969 10.1038/nri2448 19029990 PMC2724991

[B21] Gordon S Martinez FO Alternative activation of macrophages: mechanism and functions Immunity 2010 32 593 604 10.1016/j.immuni.2010.05.007 20510870

[B22] Rao S Lobov IB Vallance JE Tsujikawa K Shiojima I Akunuru S Walsh K Benjamin LE Lang RA Obligatory participation of macrophages in an angiopoietin 2-mediated cell death switch Development 2007 134 4449 4458 10.1242/dev.012187 18039971 PMC3675770

[B23] Rymo SF Gerhardt H Wolfhagen Sand F Lang R Uv A Betsholtz C A two-way communication between microglial cells and angiogenic sprouts regulates angiogenesis in aortic ring cultures PLoS One 2011 6 e15846 10.1371/journal.pone.0015846 21264342 PMC3018482

[B24] Stefater JA Lewkowich I Rao S Mariggi G Carpenter AC Burr AR Fan J Ajima R Molkentin JD Williams BO Regulation of angiogenesis by a non-canonical Wnt-Flt1 pathway in myeloid cells Nature 2011 474 511 515 10.1038/nature10085 21623369 PMC3214992

[B25] Grunewald M Avraham I Dor Y Bachar-Lustig E Itin A Jung S Chimenti S Landsman L Abramovitch R Keshet E VEGF-induced adult neovascularization: recruitment, retention, and role of accessory cells Cell 2006 124 175 189 10.1016/j.cell.2005.10.036 16413490

[B26] Hazan AD Smith SD Jones RL Whittle W Lye SJ Dunk CE Vascular-leukocyte interactions: mechanisms of human decidual spiral artery remodeling in vitro Am J Pathol 2010 177 1017 1030 10.2353/ajpath.2010.091105 20558572 PMC2913364

[B27] Moldovan NI Goldschmidt-Clermont PJ Parker-Thornburg J Shapiro SD Kolattukudy PE Contribution of monocytes/macrophages to compensatory neovascularization: the drilling of metalloelastase-positive tunnels in ischemic myocardium Circ Res 2000 87 378 384 10.1161/01.RES.87.5.378 10969035

[B28] Bertrand JY Jalil A Klaine M Jung S Cumano A Godin I Three pathways to mature macrophages in the early mouse yolk sac Blood 2005 106 3004 3011 10.1182/blood-2005-02-0461 16020514

[B29] Ginhoux F Greter M Leboeuf M Nandi S See P Gokhan S Mehler MF Conway SJ Ng LG Stanley ER Fate mapping analysis reveals that adult microglia derive from primitive macrophages Science 2010 330 841 845 10.1126/science.1194637 20966214 PMC3719181

[B30] Wigle JT Oliver G Prox1 function is required for the development of the murine lymphatic system Cell 1999 98 769 778 10.1016/S0092-8674(00)81511-1 10499794

[B31] Francois M Short K Secker GA Combes A Schwarz Q Davidson TL Smyth I Hong YK Harvey NL Koopman P Segmental territories along the cardinal veins generate lymph sacs via a ballooning mechanism during embryonic lymphangiogenesis in mice Dev Biol 2012 364 89 98 10.1016/j.ydbio.2011.12.032 22230615

[B32] Karkkainen MJ Haiko P Sainio K Partanen J Taipale J Petrova TV Jeltsch M Jackson DG Talikka M Rauvala H Vascular endothelial growth factor C is required for sprouting of the first lymphatic vessels from embryonic veins Nat Immunol 2004 5 74 80 10.1038/ni1013 14634646

[B33] Francois M Caprini A Hosking B Orsenigo F Wilhelm D Browne C Paavonen K Karnezis T Shayan R Downes M Sox18 induces development of the lymphatic vasculature in mice Nature 2008 456 643 647 10.1038/nature07391 18931657

[B34] Srinivasan RS Geng X Yang Y Wang Y Mukatira S Studer M Porto MP Lagutin O Oliver G The nuclear hormone receptor Coup-TFII is required for the initiation and early maintenance of Prox1 expression in lymphatic endothelial cells Genes Dev 2010 24 696 707 10.1101/gad.1859310 20360386 PMC2849126

[B35] Tammela T Alitalo K Lymphangiogenesis: Molecular mechanisms and future promise Cell 2010 140 460 476 10.1016/j.cell.2010.01.045 20178740

[B36] Wang Y Oliver G Current views on the function of the lymphatic vasculature in health and disease Genes Dev 2010 24 2115 2126 10.1101/gad.1955910 20889712 PMC2947764

[B37] Ny A Koch M Schneider M Neven E Tong RT Maity S Fischer C Plaisance S Lambrechts D Heligon C A genetic Xenopus laevis tadpole model to study lymphangiogenesis Nat Med 2005 11 998 1004 16116431 10.1038/nm1285

[B38] Buttler K Kreysing A von Kaisenberg CS Schweigerer L Gale N Papoutsi M Wilting J Mesenchymal cells with leukocyte and lymphendothelial characteristics in murine embryos Dev Dyn 2006 235 1554 1562 10.1002/dvdy.20737 16502417

[B39] Wilting J Aref Y Huang R Tomarev SI Schweigerer L Christ B Valasek P Papoutsi M Dual origin of avian lymphatics Dev Biol 2006 292 165 173 10.1016/j.ydbio.2005.12.043 16457798

[B40] Buttler K Ezaki T Wilting J Proliferating mesodermal cells in murine embryos exhibiting macrophage and lymphendothelial characteristics BMC Dev Biol 2008 8 43 10.1186/1471-213X-8-43 18430230 PMC2375885

[B41] Srinivasan RS Dillard ME Lagutin OV Lin FJ Tsai S Tsai MJ Samokhvalov IM Oliver G Lineage tracing demonstrates the venous origin of the mammalian lymphatic vasculature Genes Dev 2007 21 2422 2432 10.1101/gad.1588407 17908929 PMC1993873

[B42] Venneri MA De Palma M Ponzoni M Pucci F Scielzo C Zonari E Mazzieri R Doglioni C Naldini L Identification of proangiogenic TIE2-expressing monocytes (TEMs) in human peripheral blood and cancer Blood 2007 109 5276 5285 10.1182/blood-2006-10-053504 17327411

[B43] Kubota Y Takubo K Shimizu T Ohno H Kishi K Shibuya M Saya H Suda T M-CSF inhibition selectively targets pathological angiogenesis and lymphangiogenesis J Exp Med 2009 206 1089 1102 10.1084/jem.20081605 19398755 PMC2715025

[B44] Wiktor-Jedrzejczak W Bartocci A Ferrante AW Ahmed-Ansari A Sell KW Pollard JW Stanley ER Total absence of colony-stimulating factor 1 in the macrophage-deficient osteopetrotic (op/op) mouse Proc Natl Acad Sci USA 1990 87 4828 4832 10.1073/pnas.87.12.4828 2191302 PMC54211

[B45] Bohmer R Neuhaus B Buhren S Zhang D Stehling M Bock B Kiefer F Regulation of developmental lymphangiogenesis by Syk(+) leukocytes Dev Cell 2010 18 437 449 10.1016/j.devcel.2010.01.009 20230750

[B46] Polli M Dakic A Light A Wu L Tarlinton DM Nutt SL The development of functional B lymphocytes in conditional PU.1 knock-out mice. Blood 2005 106 2083 2090 10.1182/blood-2005-01-0283 15933053

[B47] Li J Chen K Zhu L Pollard JW Conditional deletion of the colony stimulating factor-1 receptor (c-fms proto-oncogene) in mice Genesis 2006 44 328 335 10.1002/dvg.20219 16823860

[B48] Baluk P Tammela T Ator E Lyubynska N Achen MG Hicklin DJ Jeltsch M Petrova TV Pytowski B Stacker SA Pathogenesis of persistent lymphatic vessel hyperplasia in chronic airway inflammation J Clin Invest 2005 115 247 257 15668734 10.1172/JCI22037 PMC544601

[B49] Kataru RP Jung K Jang C Yang H Schwendener RA Baik JE Han SH Alitalo K Koh GY Critical role of CD11b + macrophages and VEGF in inflammatory lymphangiogenesis, antigen clearance, and inflammation resolution Blood 2009 113 5650 5659 10.1182/blood-2008-09-176776 19346498

[B50] Huggenberger R Siddiqui SS Brander D Ullmann S Zimmermann K Antsiferova M Werner S Alitalo K Detmar M An important role of lymphatic vessel activation in limiting acute inflammation Blood 2011 117 4667 4678 10.1182/blood-2010-10-316356 21364190 PMC3099581

[B51] Kerjaschki D Regele HM Moosberger I Nagy-Bojarski K Watschinger B Soleiman A Birner P Krieger S Hovorka A Silberhumer G Lymphatic neoangiogenesis in human kidney transplants is associated with immunologically active lymphocytic infiltrates J Am Soc Nephrol 2004 15 603 612 10.1097/01.ASN.0000113316.52371.2E 14978162

[B52] Yin N Zhang N Xu J Shi Q Ding Y Bromberg JS Targeting lymphangiogenesis after islet transplantation prolongs islet allograft survival Transplantation 2011 92 25 30 10.1097/TP.0b013e31821d2661 21508896 PMC3703312

[B53] Zheng Y Lin H Ling S Clinicopathological correlation analysis of (lymph) angiogenesis and corneal graft rejection Mol Vis 2011 17 1694 1700 21738399 PMC3130724

[B54] Kim KE Koh YJ Jeon BH Jang C Han J Kataru RP Schwendener RA Kim JM Koh GY Role of CD11b + macrophages in intraperitoneal lipopolysaccharide-induced aberrant lymphangiogenesis and lymphatic function in the diaphragm Am J Pathol 2009 175 1733 1745 10.2353/ajpath.2009.090133 19762711 PMC2751568

[B55] Maruyama K Ii M Cursiefen C Jackson DG Keino H Tomita M Van Rooijen N Takenaka H D'Amore PA Stein-Streilein J Inflammation-induced lymphangiogenesis in the cornea arises from CD11b-positive macrophages J Clin Invest 2005 115 2363 2372 10.1172/JCI23874 16138190 PMC1193872

[B56] Maruyama K Asai J Ii M Thorne T Losordo DW D'Amore PA Decreased macrophage number and activation lead to reduced lymphatic vessel formation and contribute to impaired diabetic wound healing Am J Pathol 2007 170 1178 1191 10.2353/ajpath.2007.060018 17392158 PMC1829452

[B57] Kerjaschki D Huttary N Raab I Regele H Bojarski-Nagy K Bartel G Krober SM Greinix H Rosenmaier A Karlhofer F Lymphatic endothelial progenitor cells contribute to de novo lymphangiogenesis in human renal transplants Nat Med 2006 12 230 234 10.1038/nm1340 16415878

[B58] Maruyama K Nakazawa T Cursiefen C Maruyama Y Van Rooijen N D'Amore PA Kinoshita S The maintenance of lymphatic vessels in the cornea is dependent on the presence of macrophages Invest Ophthalmol Vis Sci 2012 53 3145 3153 10.1167/iovs.11-8010 22511631

[B59] Zhang Q Lu Y Proulx ST Guo R Yao Z Schwarz EM Boyce BF Xing L Increased lymphangiogenesis in joints of mice with inflammatory arthritis Arthritis Res Ther 2007 9 R118 10.1186/ar2326 17997858 PMC2246237

[B60] Yin N Zhang N Lal G Xu J Yan M Ding Y Bromberg JS Lymphangiogenesis is required for pancreatic islet inflammation and diabetes PLoS One 2011 6 e28023 10.1371/journal.pone.0028023 22132197 PMC3223214

[B61] Shi VY Bao L Chan LS Inflammation-driven Dermal Lymphangiogenesis in Atopic Dermatitis is Associated with CD11b+ Macrophage Recruitment and VEGF-C Up-regulation in the IL-4-Transgenic Mouse Model Microcirculationin press 10.1111/j.1549-8719.2012.00189.x 22574929

[B62] Machnik A Neuhofer W Jantsch J Dahlmann A Tammela T Machura K Park JK Beck FX Muller DN Derer W Macrophages regulate salt-dependent volume and blood pressure by a vascular endothelial growth factor-C-dependent buffering mechanism Nat Med 2009 15 545 552 10.1038/nm.1960 19412173

[B63] Lee JY Park C Cho YP Lee E Kim H Kim P Yun SH Yoon YS Podoplanin-expressing cells derived from bone marrow play a crucial role in postnatal lymphatic neovascularization Circulation 2010 122 1413 1425 10.1161/CIRCULATIONAHA.110.941468 20855662 PMC2989430

[B64] Hall KL Volk-Draper LD Flister MJ Ran S New model of macrophage acquisition of the lymphatic endothelial phenotype PLoS One 2012 7 e31794 10.1371/journal.pone.0031794 22396739 PMC3292559

[B65] Zumsteg A Baeriswyl V Imaizumi N Schwendener R Ruegg C Christofori G Myeloid cells contribute to tumor lymphangiogenesis PLoS One 2009 4 e7067 10.1371/journal.pone.0007067 19759906 PMC2738969

[B66] Schoppmann SF Birner P Stockl J Kalt R Ullrich R Caucig C Kriehuber E Nagy K Alitalo K Kerjaschki D Tumor-associated macrophages express lymphatic endothelial growth factors and are related to peritumoral lymphangiogenesis Am J Pathol 2002 161 947 956 10.1016/S0002-9440(10)64255-1 12213723 PMC1867252

[B67] Cursiefen C Chen L Borges LP Jackson D Cao J Radziejewski C D'Amore PA Dana MR Wiegand SJ Streilein JW VEGF-A stimulates lymphangiogenesis and hemangiogenesis in inflammatory neovascularization via macrophage recruitment J Clin Invest 2004 113 1040 1050 15057311 10.1172/JCI20465 PMC379325

[B68] Jeon BH Jang C Han J Kataru RP Piao L Jung K Cha HJ Schwendener RA Jang KY Kim KS Profound but dysfunctional lymphangiogenesis via vascular endothelial growth factor ligands from CD11b + macrophages in advanced ovarian cancer Cancer Res 2008 68 1100 1109 10.1158/0008-5472.CAN-07-2572 18281485

[B69] Kunstfeld R Hirakawa S Hong YK Schacht V Lange-Asschenfeldt B Velasco P Lin C Fiebiger E Wei X Wu Y Induction of cutaneous delayed-type hypersensitivity reactions in VEGF-A transgenic mice results in chronic skin inflammation associated with persistent lymphatic hyperplasia Blood 2004 104 1048 1057 10.1182/blood-2003-08-2964 15100155

[B70] Cursiefen C Maruyama K Bock F Saban D Sadrai Z Lawler J Dana R Masli S Thrombospondin 1 inhibits inflammatory lymphangiogenesis by CD36 ligation on monocytes J Exp Med 2011 208 1083 1092 10.1084/jem.20092277 21536744 PMC3092349

[B71] Ruddell A Mezquita P Brandvold KA Farr A Iritani BM B lymphocyte-specific c-Myc expression stimulates early and functional expansion of the vasculature and lymphatics during lymphomagenesis Am J Pathol 2003 163 2233 2245 10.1016/S0002-9440(10)63581-X 14633598 PMC1892400

[B72] Angeli V Ginhoux F Llodra J Quemeneur L Frenette PS Skobe M Jessberger R Merad M Randolph GJ B cell-driven lymphangiogenesis in inflamed lymph nodes enhances dendritic cell mobilization Immunity 2006 24 203 215 10.1016/j.immuni.2006.01.003 16473832

[B73] Kataru RP Kim H Jang C Choi DK Koh BI Kim M Gollamudi S Kim YK Lee SH Koh GY T lymphocytes negatively regulate lymph node lymphatic vessel formation Immunity 2011 34 96 107 10.1016/j.immuni.2010.12.016 21256057

[B74] Achen MG McColl BK Stacker SA Focus on lymphangiogenesis in tumor metastasis Cancer Cell 2005 7 121 127 10.1016/j.ccr.2005.01.017 15710325

[B75] Tammela T He Y Lyytikka J Jeltsch M Markkanen J Pajusola K Yla-Herttuala S Alitalo K Distinct architecture of lymphatic vessels induced by chimeric vascular endothelial growth factor-C/vascular endothelial growth factor heparin-binding domain fusion proteins Circ Res 2007 100 1468 1475 10.1161/01.RES.0000269043.51272.6d 17478733

[B76] He Y Rajantie I Pajusola K Jeltsch M Holopainen T Yla-Herttuala S Harding T Jooss K Takahashi T Alitalo K Vascular endothelial cell growth factor receptor 3-mediated activation of lymphatic endothelium is crucial for tumor cell entry and spread via lymphatic vessels Cancer Res 2005 65 4739 4746 10.1158/0008-5472.CAN-04-4576 15930292

[B77] Yang H Kim C Kim MJ Schwendener RA Alitalo K Heston W Kim I Kim WJ Koh GY Soluble vascular endothelial growth factor receptor-3 suppresses lymphangiogenesis and lymphatic metastasis in bladder cancer Mol Cancer 2011 10 36 10.1186/1476-4598-10-36 21481239 PMC3080348

[B78] Zhang B Wang J Gao J Guo Y Chen X Wang B Gao J Rao Z Chen Z Alternatively activated RAW264.7 macrophages enhance tumor lymphangiogenesis in mouse lung adenocarcinoma J Cell Biochem 2009 107 134 143 10.1002/jcb.22110 19241443

[B79] Schoppmann SF Fenzl A Nagy K Unger S Bayer G Geleff S Gnant M Horvat R Jakesz R Birner P VEGF-C expressing tumor-associated macrophages in lymph node positive breast cancer: impact on lymphangiogenesis and survival Surgery 2006 139 839 846 10.1016/j.surg.2005.12.008 16782443

[B80] Moussai D Mitsui H Pettersen JS Pierson KC Shah KR Suarez-Farinas M Cardinale IR Bluth MJ Krueger JG Carucci JA The human cutaneous squamous cell carcinoma microenvironment is characterized by increased lymphatic density and enhanced expression of macrophage-derived VEGF-C J Invest Dermatol 2011 131 229 236 10.1038/jid.2010.266 20827282

[B81] Algars A Irjala H Vaittinen S Huhtinen H Sundstrom J Salmi M Ristamaki R Jalkanen S Type and location of tumor-infiltrating macrophages and lymphatic vessels predict survival of colorectal cancer patients Int J Cancer 2011 131 864 873 21952788 10.1002/ijc.26457

[B82] Karnezis T Shayan R Caesar C Roufail S Harris NC Ardipradja K Zhang YF Williams SP Farnsworth RH Chai MG VEGF-D promotes tumor metastasis by regulating prostaglandins produced by the collecting lymphatic endothelium Cancer Cell 2012 21 181 195 10.1016/j.ccr.2011.12.026 22340592

